# Guiding Effect of Serum Procalcitonin (PCT) on the Antibiotic Application to Patients with Sepsis

**Published:** 2017-11

**Authors:** Yu LIU, Weize YANG, Jie WEI

**Affiliations:** Dept. of Emergency, Renmin Hospital of Wuhan University, Wuhan, Hubei Province, China

**Keywords:** Sepsis, Serum, Procalcitonin, Antibiotic

## Abstract

**Background:**

This study aimed to investigate the guiding effect of serum procalcitonin (PCT) on the antibiotic application to patients with sepsis.

**Methods:**

Ninety-eight patients with sepsis treated in Renmin Hospital of Wuhan University, China from 2015–2017 were enrolled. They were divided into two equal groups of control group and the observation group. Patients in the observation group received the antibiotic therapy guided by PCT while patients in the control group received the regular antibiotic therapy. The conventional indexes, prognosis and clinical effects of the two groups were compared.

**Results:**

There were no statistical significance in the differences of the Acute Physiology and Chronic Health Evaluation II (APACHE-II) scores C-reactive protein (CRP) and white blood cell count (WBC) levels between the two groups. The duration of the antibiotic application to patients in the observation group was (7.74±0.61) d which was (10.22±0.78) d shorter than that to patients in the control group. The difference was statistically significant (*P<*0.05). The Intensive Care Unit (ICU) stay time and the hospital stays of patients in the observation group were shorter than those of patients in the control group. The difference had statistical significance (*P<*0.05). The difference in 30-day-recurrence rate and 30 day-mortality of the patients in the two groups had no statistical significance. There was statistically significant difference in the comparison of the clinical effects between the two groups.

**Conclusion:**

Guiding effects of the serum PCT on the application of antibiotics to patients with sepsis shorten the usage time of the antibiotics, ICU stay time and hospital stays.

## Introduction

The sepsis is a systemic inflammatory response syndrome caused by infections, which is a common intensive care unit disease. The mortality of patients with sepsis is about 18%–50% because of multiple organ failure. Therefore, it is necessary to conduct actively rational anti-infective therapies to patients with sepsis in clinic (1). In clinic, the inflammatory indexes, including the white blood cell count and C-reactive protein, are usually adopted to evaluate the conditions of patients with sepsis which however, lack specificity of the early-stage diagnosis since the specificity and sensitivity of these indexes are not high (2).

Procalcitonin (PCT) is a kind of calcitonin propeptide matter without the activity of hormone which can react to the severity of bacterial infection and its control status. As a result, the specificity and sensitivity of PCT are better than that of traditional clinical inflammatory indexes (3). For patients with lower respiratory infection, when the serum PCT is over 0.25μg/L, the bacterial infection exists (3, 4). Then, the antibiotic therapy could be conducted for patients with lower respiratory infection and the antibiotic dosage could be effectively decreased based on PCT level.

We investigated the guiding effect of serum PCT on the antibiotic application to patients with sepsis to provide a reference for later clinical treatment.

## Methods

### Patients

This study was approved by the Ethical Committee of Renmin Hospital of Wuhan University, and signed written informed consents were obtained from the patients and/or guardians.

Ninety-eight patients with sepsis treated in Renmin Hospital of Wuhan University, China from January 2015 to January 2017 were enrolled. They were divided into the control group and the observation group with 49 patients in each group by the random number table. In the observation group, there were 27 male patients and 22 female patients. The age range of the patients was between 18 and 79 and the average age was (66.38±9.32) yr old. There were 9 patients with diabetes, 17 patients with hypertension, 7 patients with coronary heart disease and 5 patients with renal insufficiency. In the control group, there were 29 male patients and 20 female patients. The age range of the patients was between 19 and 80 and the average age was (65.22±10.13) yr old. There were 10 patients with diabetes, 15 patients with hypertension, 9 patients with coronary heart disease and 4 patients with renal insufficiency. The difference in the general information of the two groups had no statistical significance (*P>*0.05) which could be compared.

Inclusion criteria: 1. Patients with bacteria-infected sepsis diagnosed in clinic; 2. Patients who was no less than 18 years old; 3. The Intensive Care Unit (ICU) stay time was no more than 72 hours; 4. Patients voluntarily signed the informed consent.

Exclusion criteria: 1. Patients who preventatively took the antibiotic; 2. Patients who once took the antibiotics due to the virus infection and bacterial infection; 3. Patients with malignant tumor; 4. Patients who were not estimated to survive and leave hospital.

### Assays

Before receiving the antibiotic therapy, patients in both groups had received the blood culture examination. The bacterial culture was performed based on the possible infection sites. The initial usage of the antibiotics for all patients should conform to the antibacterial agent selection principle of sepsis. The serum PCT levels of patients in the observation group were monitored every day until 3d after the patients stopped taking antibiotics. Patients whose serum PCT level decrease exceeded the peak value 90% or the absolute values of PCT were no more than 0.25μg/L stopped taking the antibiotics.

### Observation indexes

The conventional indexes, prognosis and clinical effects of patients in the two groups were compared. 1. The conventional indexes: the Acute Physiology and Chronic Health Evaluation II (APACHE-II) scores, C-reactive protein (CRP) and white blood cell count (WBC) levels and the antibiotic usage time; 2. The prognosis: ICU stay time, hospital stays, 30 day-recurrence rate and 30-day mortality; 3. Clinical effects: recovery, improvement and treatment abandoning.

### Statistical methods

SPSS 20.0 (Chicago, IL, USA) was adopted to conduct the statistical analysis and *t*-test and χ^2^ test were used for measurement data and enumeration data respectively. *P<*0.05 suggested that the difference was statistically significant.

## Results

### Comparisons of the conventional indexes

The difference in APACHE-II scores, CRP and WBC levels of the two groups had no statistical significance. The antibiotic usage time of patients in the observation group was (7.74±0.61) d which was (10.22±0.78) d shorter than that of patients in the control group. The difference had statistical significance (*P<*0.05) (Table 1).

**Table 1:** Comparisons of the conventional indexes

***Group***	***Case (n)***	***APACHE-II scores (point)***	***CRP(mg/L)***	***WBC(×109/L)***	***Antibiotic usage time (d)***
Observation group	49	80.43±8.25	14.25±5.14	11.91±8.72	7.74±0.61
Control group	49	83.72±8.84	12.74±5.77	10.82±6.03	10.22±0.78
t value		1.697	1.945	1.637	2.678
P value		0.086	0.057	0.112	0.009

### Comparisons of the prognosis

ICU stay time and hospital stays of patients in the observation group were shorter than those of patients in the control group. The difference had statistical significance (*P<*0.05). The difference in 30-day recurrence rate and 30 day-mortality of patients in both two groups had no statistical significance (Table 2).

**Table 2:** Comparisons of the prognosis

***Group***	***Case (n)***	***ICU stay time (d)***	***Hospital stays(d)***	***30d-recurrence rate (n, %)***	***30d-mortality (n, %)***
Observation group	49	10.55±3.43	19.78±5.43	3 (6.12)	5 (10.20)
Control group	49	13.97±3.76	23.17±6.22	2 (4.08)	6 (12.24)
χ^2^/t value		2.147	2.509	2.539	1.653
P value		0.040	0.021	0063	0.081

### Comparisons of clinical effects

The difference in clinical effects between the two groups had statistical significance (*P<*0.001) (Table 3).

**Table 3:** Comparisons of clinical effects (n (%))

***Group***	***Case (n)***	***Recovery***	***Improvement***	***Treatment abandoning***
Observation group	49	15 (30.61)	29 (59.19)	5 (10.20)
Control group	49	13 (26.53)	31 (63.27)	5 (10.20)
χ^2^/t value		1.976	1.627	2.987
P value		0.070	0.083	0.059

## Discussion

The sepsis is a common ICU disease. The early diagnosis and the antibiotic drug therapy for patients with sepsis are very important to help patients obtain the good prognosis. However, the inappropriate antibiotic therapy can often result in drug-resistance bacteria, aggravating the disease (5). Currently, the clinical indexes, including the body temperature, etiology and blood routine, are examined to evaluate the infection conditions of patients. However, because of the long inspection time and low sensitivity and specificity of these indexes, it is necessary to adopt the index that has higher sensitivity and specificity to guide the usage of the antibiotics to patients in clinic to reduce relevant adverse reactions due to the inappropriate usage of the antibiotic (6, 7).

PCT is a new-type inflammatory index, a kind of calcitonin propeptide matter without the activity of hormone and a sepsis-induced protein. Physically, PCT is produced by Thyroid C cell, which almost cannot be detected in the serum of healthy people. However, if patients are infected by the bacteria, PCT level in the blood will be increased because PCT mainly comes from other organs except the thyroid and the bacteria factors or endotoxin can suppress the decomposition course of PCT, which is then released into the blood (8–12). Therefore, this paper investigated the guiding effect of serum PCT on the antibiotic application to patients with sepsis to provide a reference for later clinical treatment.

Currently, the biological functions of PCT are not completely and clearly identified in clinic. Usually, PCT is known as a kind of non-steroidal anti-inflammatory matter, which plays an important role in regulating the cytokine network. PCT level will be increased rapidly after being induced. After the infection, PCT level in the serum is increased in a short time which usually can be increased within 2–6 hours after the infection and reach to the peak value 12 hours after the infection. PCT has a good stability, which will not be affected by the environment in vitro and vivo, such as the temperature. It is also not easy to degrade and has a 24-hour half-life period. In addition, by comparing with other inflammatory mediums, the testing methods of PCT level in clinic are reliable and simple and consume less time (10). When patients were infected by the bacterial, the serum PCT level was increased significantly. However, when patients were infected by the virus or even serious virus, the serum PCT level changed or increased slightly.

The results of this paper showed that the difference of APACHE-IIscores and CRP and WBC levels had no statistical significance. The antibiotic usage time of patients in the observation group was (7.74±0.61) d which was (10.22±0.78) d shorter than that of patients in the control group. The difference had statistical significance (*P=*0.009). ICU stay time and hospital stays of patients in the observation group were shorter than that of patients in the control group. The difference had statistical significance. The difference in 30 day-recurrence rate and 30 day-mortality of the patients between the two groups had no statistical significance (*P=*0.063). The results show that the serum PCT can guide clinical physicians to evaluate the infection conditions of patients with sepsis and guide the antibiotic usage to avoid the antibiotics abuse, standardize the antibiotics use, shorten the usage time of the antibiotics, improve the conditions of patients and shorten the recovery time. However, the nutriture conditions of severe patients or old patients with sepsis are not good in the clinical study, the patients have low reactivity to the stimulation and the serum PCT level is not high. As a result, it is necessary to combine the guiding function of PCT with the clinical evaluation and the comprehensive assessments, including signs, symptoms and auxiliary examinations of patients, to decide the application of the antibiotics.

## Conclusion

The guiding effects of the serum PCT on the application of antibiotics to patients with sepsis can shorten the usage time of the antibiotics, ICU stay time and hospital stays, which will not affect the prognosis of patients and can be further applied in clinic.

## Ethical considerations

Ethical issues (Including plagiarism, informed consent, misconduct, data fabrication and/or falsification, double publication and/or submission, redundancy, etc.) have been completely observed by the authors.
